# Copper Affects Composition and Functioning of Microbial Communities in Marine Biofilms at Environmentally Relevant Concentrations

**DOI:** 10.3389/fmicb.2018.03248

**Published:** 2019-01-08

**Authors:** Natàlia Corcoll, Jianghua Yang, Thomas Backhaus, Xiaowei Zhang, Karl Martin Eriksson

**Affiliations:** ^1^Department of Biological and Environmental Sciences, University of Gothenburg, Gothenburg, Sweden; ^2^State Key Laboratory of Pollution Control and Resource Reuse, School of the Environment, Nanjing University, Nanjing, China; ^3^Department of Mechanics and Maritime Sciences, Chalmers University of Technology, Gothenburg, Sweden

**Keywords:** metabarcoding, 16S, 18S, periphyton, amplicon-sequencing, metals

## Abstract

Copper (Cu) pollution in coastal areas is a worldwide threat for aquatic communities. This study aims to demonstrate the usefulness of the DNA metabarcoding analysis in order to describe the ecotoxicological effect of Cu at environmental concentrations on marine periphyton. Additionally, the study investigates if Cu-induced changes in community structure co-occurs with changes in community functioning (i.e., photosynthesis and community tolerance to Cu). Periphyton was exposed for 18 days to five Cu concentrations, between 0.01 and 10 μM, in a semi-static test. Diversity and community structure of prokaryotic and eukaryotic organisms were assessed by 16S and 18S amplicon sequencing, respectively. Community function was studied as impacts on algal biomass and photosynthetic activity. Additionally, we studied Pollution-Induced Community Tolerance (PICT) using photosynthesis as the endpoint. Sequencing results detected an average of 9,504 and 1,242 OTUs for 16S and 18S, respectively, reflecting the high biodiversity of marine periphytic biofilms. Eukaryotes represent the most Cu-sensitive kingdom, where effects were seen already at concentrations as low as 0.01 μM. The structure of the prokaryotic part of the community was impacted at slightly higher concentrations (0.06 μM), which is still in the range of the Cu concentrations observed in the area (0.08 μM). The current environmental quality standard for Cu of 0.07 μM therefore does not seem to be sufficiently protective for periphyton. Cu exposure resulted in a more Cu-tolerant community, which was accompanied by a reduced total algal biomass, increased relative abundance of diatoms and a reduction of photosynthetic activity. Cu exposure changed the network of associations between taxa in the communities. A total of 23 taxa, including taxa within Proteobacteria, Bacteroidetes, Stramenopiles, and Hacrobia, were identified as being particularly sensitive to Cu. DNA metabarcoding is presented as a sensitive tool for community-level ecotoxicological studies that allows to observe impacts simultaneously on a multitude of pro- and eukaryotic taxa, and therefore to identify particularly sensitive, non-cultivable taxa.

## Introduction

Copper (Cu) pollution in coastal areas is mainly associated with domestic and industrial activities (Parks et al., 2010; Oursel et al., 2013; Misson et al., 2016), and the use of Cu-based antifouling paints on ship hulls (Yebra et al., 2004; Thomas and Brooks, 2010), especially after the ban of tributyltin (TBT) in the late 1980s in France (Alzieu, 2000) and from 2003 to the rest of Europe (Yebra et al., 2004). Elevated Cu levels can be detected in many parts of the world, especially near enclosed harbors and marinas. For instance, concentrations as high as 0.33 μM have been detected in San Diego Bay, USA (Schiff et al., 2007) or up to 0.41 μM in Toulon Bay, France (Briand et al., 2017). On the west cost of Sweden, Cu levels have been detected at concentrations up to 5 μg/L (0.08 μM) (Egardt et al., 2018), exceeding the environmental quality standard (EQS) for this region, i.e., the Kattegat sea where an EQS of 4 μg/L (0.07 μM) has been established (HVMFS 2015:4). Cu is an essential element (Festa and Thiele, 2011), but becomes toxic at higher concentrations (Amara et al., 2018), depending on metal speciation, accumulation (Meylan et al., 2004; Serra et al., 2009) and exposed organism (Barranguet et al., 2003; Amara et al., 2018). In photosynthetically active cells Cu inhibits CO_2_ fixation and PSII activity (Pandey et al., 1992), causes oxidative stress and ultimately inhibits cell growth (Gonçalves et al., 2018). In bacteria, Cu affects various cellular enzymes and proteins involved in energy metabolism (Waldron et al., 2009). Cu affects species composition in microbial communities, leading to a replacement of sensitive taxa with tolerant ones (Gustavson et al., 1999; Serra et al., 2009; Ancion et al., 2010). Detailed descriptions of Cu-sensitive and -tolerant taxa in environmental communities are currently lacking.

Periphyton, also called microhytobenthos, forms biofilms of highly diverse microbial communities—including algae, bacteria, fungi, and meiofauna—that live attached to submerged substrata in aquatic ecosystems (Lock, 1993; Salta et al., 2013; Sanli et al., 2015). The importance of periphyton to aquatic ecosystems is linked to its function as a primary producer and its contribution to biogeochemical cycles (Battin et al., 2003; Sundbäck et al., 2004). The use of periphyton for studies in community ecotoxicology is well-established (Sabater et al., 2007; Eriksson et al., 2009a; Corcoll et al., 2015), as it allows to assess effects of contaminants across different levels of biological organization (Guasch et al., 2016). In this line, an approach commonly used for detecting long-term effects of toxicants in periphyton communities is the measurement of PICT (Pollution-Induced Community Tolerance), introduced by Blanck et al. (1988). PICT is based on the elimination of micro-organisms sensitive to the toxicant in question and the induced inter- and intraspecific selection for organisms that are more tolerant to the toxicant. The entire community is restructured and finally displays an overall increase in its tolerance to the toxicant, compared to an unexposed reference community. This induced tolerance is commonly quantified as an increase of the short-term EC50 of the whole community to the toxicant in question, which is perceived as a community trait (Blanck et al., 1988; Corcoll et al., 2014; Tlili et al., 2015). The use of PICT for detecting effects from Cu on marine and freshwater periphyton has shown to be more sensitive than traditional community composition based-tools such as microscope observations, pigment-profile based approaches or PCR-DGGE fingerprints (Gustavson et al., 1999; Barranguet et al., 2003; Massieux et al., 2004; Serra et al., 2009; Tlili et al., 2010).

Recent advances in DNA sequencing represents a powerful tool to detect and quantify effects of toxic substances on ecological communities with high sample/observation throughput (Zhang et al., 2009; Yang et al., 2018). In particular DNA metabarcoding, a high throughput DNA-based amplicon sequencing technique, has emerged as a new molecular tool to identify a large proportion of the biological community present in an environmental (Hebert et al., 2003). New computational methods applied to high-throughput DNA sequencing data have allowed the development of co-occurrence network analysis to explore potential interactions between taxa (e.g., inter-taxa correlations). These new analytical methods have permitted to move beyond classical focus on single properties of the microbial communities (e.g., community composition and diversity classically determined by α-diversity or ß-diversity indices; Barberán et al., 2012). For instance, in the metabarcoding work of Mandakovic et al. (2018), network analyses were applied to reveal changes in the structure and co-occurrence patterns in soil microbial communities under different environmental stress factors.

The main goal of this study is to demonstrate the usefulness of the DNA metabarcoding analysis in order to describe the ecotoxicological effect of Cu at environmental concentrations on marine periphytic biofilms. For this purpose, metabarcoding was used in order to describe the effect of Cu on the structure of the prokaryotic and eukaryotic community (i.e., biodiversity, community composition, identification of sensitive/tolerant taxa, and community network). Additionally, the study aims to investigate if Cu-induced changes in community structure co-occurs with changes in community functioning (i.e., photosynthesis and community tolerance to Cu). For these purposes, natural marine periphyton was exposed to a range of Cu concentrations (0.01–10 μM) in a semi-static microcosm for 18 days. We selected the 16S rRNA (V3 region) and the 18S rRNA (V9 region) genes to target prokaryotes and eukaryotes, respectively. Our results provide new information on how Cu pollution affects the structure and functioning of marine microbial communities, which can aid the setting of appropriate environmental quality standards.

## Materials and Methods

### Microcosm Setup and Experimental Design

The experiment was conducted indoors in a thermo-constant room at the facilities of the Sven Lovén Centre for marine sciences—Kristineberg by the Gullmar fjord on the west coast of Sweden, from 18th August to 6th September 2015. Eighteen independent microcosms made by rectangular glass vessels inspired by the SWIFT periphyton test described by Porsbring et al. (2007) were used for the experiment. Each microcosm contained 300 mL of natural sea water collected from a nearby pristine bay (Gåseviken: 58.245373°N, 11.433628°E). The sea water, with its naturally occurring organisms, was filtered through a 200 μm mesh to remove large organisms and was enriched with 0.7 μM phosphate (as KH_2_PO_4_) and 0.8 μM nitrate (as NH_4_NO_3_) to avoid nutrient limitation during periphyton growth. Periphyton was allowed to colonize rectangular polyethylene terephtalate glycol (PETG) slides (6.9 × 1.4 cm^2^). Each rectangular microcosm had a glass rod attached along the long side in the middle of the bottom of the vessel and 22 PETG slides were placed from the bottom glass rod to the side walls of the vessel, making an angle of ~22° between the bottom and the walls of the vessel. The sea water covered half of the surface of the slides. The water from each microcosm was renewed every day. To stimulate periphyton colonization and growth in the beginning of the experiment, marine periphyton inocula were prepared by brushing off periphyton from the upper part of 50–60 stones and pebbles, collected at a maximal depth of 60 cm, into seawater. The water, stones and pebbles were sampled from the same pristine bay as the natural sea water. The inocula was vigorously shaken and filtered through a 200 μm mesh to remove large organisms. Twenty milliliters of inocula, with an approximate chlorophyll *a* concentration of 0.3 μg mL^−1^, were provided twice to each microcosm during the first week of the experiment. The microcosms were incubated in a thermo-controlled room with the temperature set to 15°C. The daily light/dark cycle of 14/8 h was simulated with OSRAM FLUORA light tubes with a light intensity at the surface of the microcosms of approximately 120 μmol photons m^−2^ s^−1^. The microcosms were in constant agitation by using horizontal shakes.

The experimental design included unexposed control microcosms and 5 Cu exposure levels, each in triplicate microcosms. The nominal Cu exposure levels were: 0.01, 0.06, 0.32, 1.78, and 10 μM. Cu stocks, 1,000 times more concentrated than the nominal concentrations, were prepared from the reagent CuCl_2_·2H_2_O (CAS number: 10125-13-0, Sigma-Aldrich) with deionized water. Three hundred microliters of the CuCl_2_·2H_2_O stocks were added to the Cu microcosms, and the same volume of deionized water was added to the unexposed controls, to give the final volume of 300 mL. Cu and deionized water were introduced at the same time as the periphyton inoculum to the Cu treatments and the controls, respectively. Water temperature, pH, oxygen, and salinity was monitored periodically, at least 10 times through the experiment, using portables multi-probes (HANNA Instruments). Water of all microcosms was sampled 3 times before and after a water renewal for Cu analysis. For this, 50 mL of water were filtered through 0.45 μm, preserved with HNO_3_ (65% suprarpure) at final concentration of 1% and kept at 4°C until further analysis with ICP-MS.

### Periphyton Sampling

After 18 days, periphyton was sampled to analyse chlorophyll *a* concentration, photosynthetic pigments, photosynthetic activity, community tolerance to Cu and microbial composition of prokaryotes and eukaryotes. For each microcosm, a periphyton slurry was produced by scraping off the periphyton from the slides into 150 mL of sea water, filtered through 0.2 μm and amended with the same amount of nutrients as used in the microcosms. Five milliliters of periphyton slurry were filtered through Whatman GF/F filters and used immediately for chlorophyll *a* analyses. Ten milliliters of periphyton slurry were filtered through Whatman GF/F filters, frozen at −20°C and stored until pigments extraction. Ten milliliters of periphyton slurry was aliquoted in tubes, pelleted by centrifuged at 6500 g for 10 min at room temperature, the supernatant was removed and the resulting pellets were snap-frozen in liquid nitrogen and stored at −80°C until DNA extraction. The remaining periphyton slurry was used to determine photosynthetic activity (^14^C-incorporation) and tolerance measurements following the PICT approach. Analyses of chlorophyll *a*, pigment profiles and microbial composition were done for all treatments. For logistic reasons, photosynthetic and community tolerance measurements were only done for the control microcosms and the microcosms with a Cu exposure of 0.32 and 1.78 μM Cu.

### Chlorophyll a Concentration and Photosynthetic Pigments

Chlorophyll *a* was extracted with 10 mL of ethanol (96%) for 24 h in the dark, at room temperature, and was quantified fluorometrically (10-AU Turner fluorometer; Turner designs, Sunnyvale California, USA) according to Jespersen and Christoffersen (1987). Photosynthetic pigments were extracted in a 4 mL mixture of acetone/methanol (80%/20%, v/v) while sonicated in an ice slurry for 3 min. Ninety microliters of the extracts were filtered onto 0.45 μm filters (VWR International Syringe filters) and analyzed with high performance liquid chromatography (HPLC; Shimadze Prominence HPLC Systems) following Torstensson et al. (2015). A total of 10 photosynthetic pigments were identified and were expressed as the ratio between the peak area of each identified pigment and the peak area of Chlorophyll *a*. Fucoxanthin was used as pigment marker for the algal group of Bacillarophyta, also known as diatoms (Roy et al., 2011).

### Pollution-Induced Community Tolerance (PICT)

Pollution-Induced Community Tolerance (PICT) was quantified as increase in EC50 values, determined in short-term photosynthesis inhibition tests using ^14^C-incorporation as endpoint according to Eriksson et al. (2009b) with some modifications. From each of the studied microcosms, triplicate unexposed control samples were prepared by mixing 1 mL of periphyton slurry and 1 mL sea water. One sample from each microcosm was exposed to 0.32, 1.35, 5.66, 23.8, and 100 μM Cu by mixing 1 mL of periphyton slurry with 1 mL of Cu solutions. The sea water used for the controls and the Cu solutions was filtered through 0.2 μm and amended with the same amount of nutrients as used in the microcosms. The samples were mixed in scintillation vials and were incubated at 15°C and 120 μmol photons m^−2^ s^−1^, while gently shaken during the incubation. After 1 h, 50 μL ^14^C-sodium bicarbonate solution, with a radioactivity of 80 μCi/ml, was added to each sample. After another hour, 100 μL formaldehyde was added to each sample to terminate photosynthetic activity. The samples were acidified with 100 μL of HCl to drive off unincorporated ^14^C and 3 mL of Instagel Plus was added to each sample. Disintegrations per minute (DPM) were calculated from counts per minute (CPM) based on the correction factors for the sample quench characteristics and the machine efficiency, using a liquid scintillation spectrometer (LS 500 Beckman). The abiotic ^14^C-incorporation was estimated by adding 100 μL formaldehyde to one sample before the incubation with ^14^C-sodium bicarbonate, and the radioactivity of that sample was subtracted from the radioactivity of the other samples from the same microcosm. ^14^C-incorporation was used as an estimate of periphyton photosynthetic activity and was expressed in relative changes of disintegrations per minute (DPM). Community tolerance was determined as differences in EC_50_ values based on photosynthetic activity between unexposed control microcosms and microcosms exposed to 0.32 and 1.78 μM Cu.

### DNA Extraction, PCR Amplification, and Sequencing

Microbial composition of prokaryotes and eukaryotes was assessed by DNA metabarcoding. Total genomic DNA was extracted using the Power Biofilm® DNAIsolation Kit (MoBio Laboratories, USA) following recommendations in Corcoll et al. (2017). DNA was precipitated with sodium acetate and ethanol prior downstream analyses. Bacterial 16S rRNA genes (V3 region) and eukaryotic 18S rRNA genes (V9 region) were amplified using V3 primers (modified primers 341F and 518R) (Klindworth et al., 2013) and V9 primers (1380F and 1510R) (Amaral-Zettler et al., 2009), respectively. Triplicate PCR reactions were performed for each sample to minimize potential PCR bias. The PCR amplicon libraries were sequenced using the Ion Torrent Proton technology according to the manufacturer's protocols.

### Bioinformatics

The QIIME v.1.8.0 (Quantitative Insights Into Microbial Ecology) pipeline was used to process the raw sequences (Caporaso et al., 2010). Low quality sequences were trimmed via the “split_libraries.py” script with “-w 50 -q 20.” PCR chimera filtering was performed via “parallel_identify_chimeric_seqs.py” script in QIIME with the default parameter. Operational taxonomic units (OTUs) were selected with a sequence similarity cut-off of 97% following the UPARSE pipeline (Edgar, 2013). For each OTU, a representative sequence was chosen and taxonomy was assigned using the RDP classifier (Wang et al., 2007) against the Greengenes database (DeSantis et al., 2006) and SILVA database (Pruesse et al., 2007) for prokaryote and eukaryote community, respectively. So far there are no consensus taxonomic hierarchy for eukaryotic organisms (https://www.arb-silva.de/projects/eukaryotic-taxonomy/). In particular, some taxon does not have clear taxonomic information (e.g., class, order, family, etc). In some groups there are intermediate taxonomic levels, not present in other groups, like superphylum, subphylum or infraphylum. The database contains tens of thousands of taxa and it's very difficult to correct the classification of taxa one by one. Hence, the original classification according to SILVA was used directly in this study for eukarytoic organisms. The raw data have been deposited to the NCBI short read archive (SRA) with BioProject ID PRJNA496374, https://www.ncbi.nlm.nih.gov/Traces/study/?acc=PRJNA496374.

### Statistical Analyses

One-way ANOVAs were used to assess differences between the treatments for Chlorophyll *a* concentration, photosynthetic activity and EC_50_ values using R (R Core Team, 2013). The effective concentration of Cu that had a 50% of effect (EC_50_) was determined after fitting ^14^C-incorporation values to a dose-response model (Weifull fit) using the package “drc” (Ritz et al., 2015) in R. Principal component analysis (PCA) was employed to observe difference in microbial composition of prokaryotes and eukaryotes between the Cu treatments using weighted Unifrac distance data in QIIME (Caporaso et al., 2010). The Kaiser-Guttmann criterion was used to determine the significance of each axis of the PCA analyses. Because the Unifrac matrix includes the phylogenetic information, PCA based on the Unifrac distances can better reflect community differences than that based on taxa abundance data at family or class level. Differences in prokaryotic and eukaryotic community composition between Cu treatments were assessed by MANOVA of Unifrac distances. Differences between Cu treatments were assessed using ANOVA with Dunnett's *post-hoc* test. Correlation networks were generated by SparCC with 100 bootstraps to assign two-sided pseudo *p*-values (Friedman and Alm, 2012). For each treatment, 3 replicates were used to calculate the correlation Rho. The networks were filtered by correlation magnitudes >0.6 and < -0.6 which indicating strong co-abundance and co-exclusion relationships. The networks were visualized by Cytoscape v 3.6.1 and the topological parameters of networks were computed by NetworkAnalyzer 2.7 (Assenov et al., 2008). Topological parameters obtained were, nodes: OTUs number of networks, network density: how densely the network is populated with edges (self-loops and duplicated edges are ignored), network heterogeneity: tendency of a network to contain hub nodes, network centralization: distribution of network density, characteristic path length: expected distance between two connected nodes, avg. number of neighbors: average connectivity of a node in the network. The response of each taxon to Cu exposure was modeled with a 3-parameter log-logistic model and the 50% effects concentration (EC50) was calculated. Beta diversity was estimated by computing weighted UniFrac distances between samples (Lozupone and Knight, 2007). All samples were rarefied at the lowest sequencing depth to reduce biases resulting from differences in sequencing depth (186400 and 112730 for eukaryote and prokaryote community, respectively).

## Results

Periphyton responses after 18 days of exposure to five Cu concentrations, between 0.01 and 10 μM, in a semi-static test, are presented below.

### Experimental Conditions

Temperature, salinity, and pH were constant over the entire experiment, varying by just 1–3% between daily water renewals. Average salinity was 20.8 PSU, water temperature was 17.9°C and pH was 8.1 (*n* = 59). Cu concentrations in the controls and the samples with nominal concentration 0.01 μM Cu were below the quantification limit of 0.02 μM. For the rest of the samples, the analyzed Cu was between 33 % and 90 % of the nominal concentrations, being closer to the nominal concentrations at higher exposure levels (Supplementary Table 1). Therefore, the nominal concentrations are used to describe the concentration response patterns.

### Effects on Photosynthetic Pigments, Community Tolerance Development, and Photosynthetic Activity

**Figure 1:**
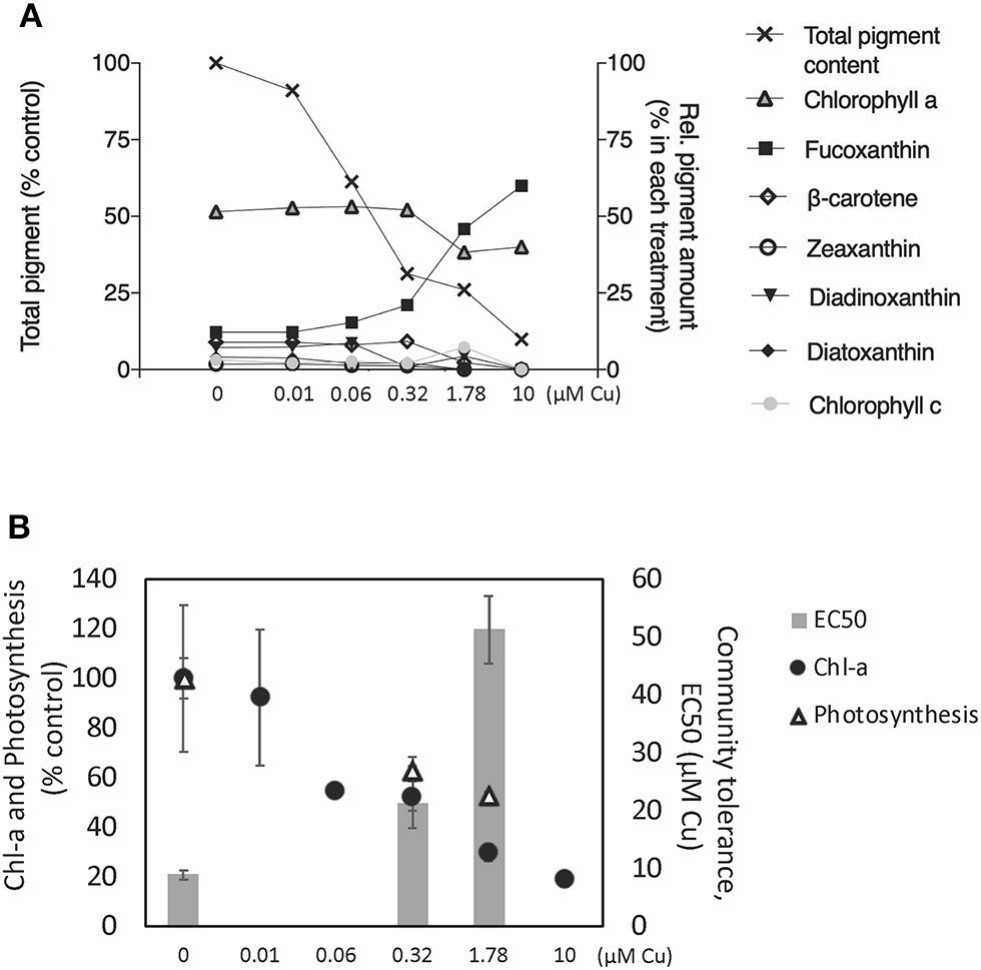
**(A)** Pigment profiles (HPLC analyses) presented as relative abundance of each pigment (right Y-axis) and sum of all pigments (left Y-axis), **(B)** Chlorophyll *a* (Chl-a) concentration (fluorometric analyses) and photosynthetic activity in percentage of the control is showed in the left Y-axis and community tolerance to Cu exposure is showed in the right Y-axis. All values represent AVG ± SE; *n* = 3 except in **(B)** where the EC50 for 1.78 was AVG ± SE; *n* = 2.

In the unexposed controls, chlorophyll *a* accounted for 52%, fucoxanthin for 12%, diadionoxanthin + diatoxanthin for 11%, ß-carotens for 9%, chlorophyll c for 3%, zeaxanthin for 2% and the remaining three non-identified pigments accounted for 11% of the total pigments (arithmetic mean of three replicates) (Figure 1A). Chlorophyll *a* concentration decreased in a concentration-dependent manner, reaching 81% inhibition at the highest concentration of 10 μM (Figure 1B). A similar pattern was observed for the total pigment content of the periphyton (Figure 1A). In contrast, the relative abundance of fucoxanthin increased in a concentration-dependent manner, increasing up to 60% of the total pigment content in the highest Cu exposure, 10 μM Cu (Figure 1B). Photosynthetic activity in all the short-term PICT detection tests was inhibited by increasing Cu concentrations, and EC50 values could be determined for all microcosms except one of the microcosms exposed to 1.78 μM Cu. The short-term EC50 (AVG ± SE) of the untreated controls was 8.88 ± 0.78 μM. After pre-exposure to 0.32 μM Cu the short-term EC50 increased to 21.3 ± 1.34 μM and after pre-exposure to Cu at 1.78 μM it increased further to 51.24 ± 5.85 μM (Figure 1B). That is, Cu pre-exposure increased the tolerance of the community to short-term exposure by being 2.3 and 5.7, respectively, higher than in control treatment indicating an increase of community tolerance to copper exposure. The photosynthetic activity (^14^C- incorporation) at concentrations of 0.32 and 1.78 μM C was inhibited up to 60% (Figure 1B).

### Effects on Prokaryotic and Eukaryotic Community Composition

DNA-sequencing of the 16S and 18S gene fragments yielded a total of 7,109,298 and 5,655,641 high quality reads, respectively. These reads clustered into 17,445 prokaryotic OTUs and 2,151 eukaryotic OTUs (Table 1). As shown by the Chao1 diversity index (Chao, 1984; Supplementary Figure 1) the sequencing depth was sufficient to achieve the saturation point for identifying both prokaryote and eukaryote taxa.

**Table 1:** Sequencing information for each sample.

	**Prokaryote**				**Eukaryote**			
**Cu (μM)**	**Raw sequences**	**High quality reads**	**OTUs**	**Core OTUs**	**Raw sequences**	**High quality reads**	**OTUs**	**Core OTUs**
0	401,492	352,513	10,673	7,343	298,545	287,308	1,359	1,048
0	421,254	370,057	10,598		314,776	304,546	1,298	
0	529,896	463,714	10,729		371,406	355,285	1,372	
0.01	499,314	437,519	10,842	7,137	397,999	380,767	1,446	1,095
0.01	481,209	428,187	10,355		367,233	353,131	1,353	
0.01	485,444	435,963	10,052		333,989	321,285	1,429	
0.06	520,732	458,352	10,448	7,354	351,999	338,684	1,398	1,211
0.06	445,386	386,729	10,675		308,046	295,090	1,378	
0.06	539,309	470,684	10,615		405,241	389,808	1,498	
0.32	553,859	493,324	9,830	6,802	353,213	339,263	1,340	1,037
0.32	594,508	531,872	9,925		409,692	395,912	1,333	
0.32	354,786	312,126	9,953		250,921	241,191	1,334	
1.78	392,169	353,045	7,214	4,537	321,938	312,893	1,138	742
1.78	385,956	352,677	6,781		347,776	338,072	1,033	
1.78	357,418	326,682	6,934		327,889	319,083	1,070	
10	236,199	209,893	8,161	5,137	160,531	155,115	856	558
10	500,776	445,464	7,994		323,477	314,640	891	
10	310,783	280,497	7,514		220,522	213,568	838	
Total	8,010,490	7,109,298	17,445	11,549	5,865,193	5,655,641	2,151	1,618

*Core OTUs means the OTU was detected by all of the three samples in the same treatment*.

**Figure 2:**
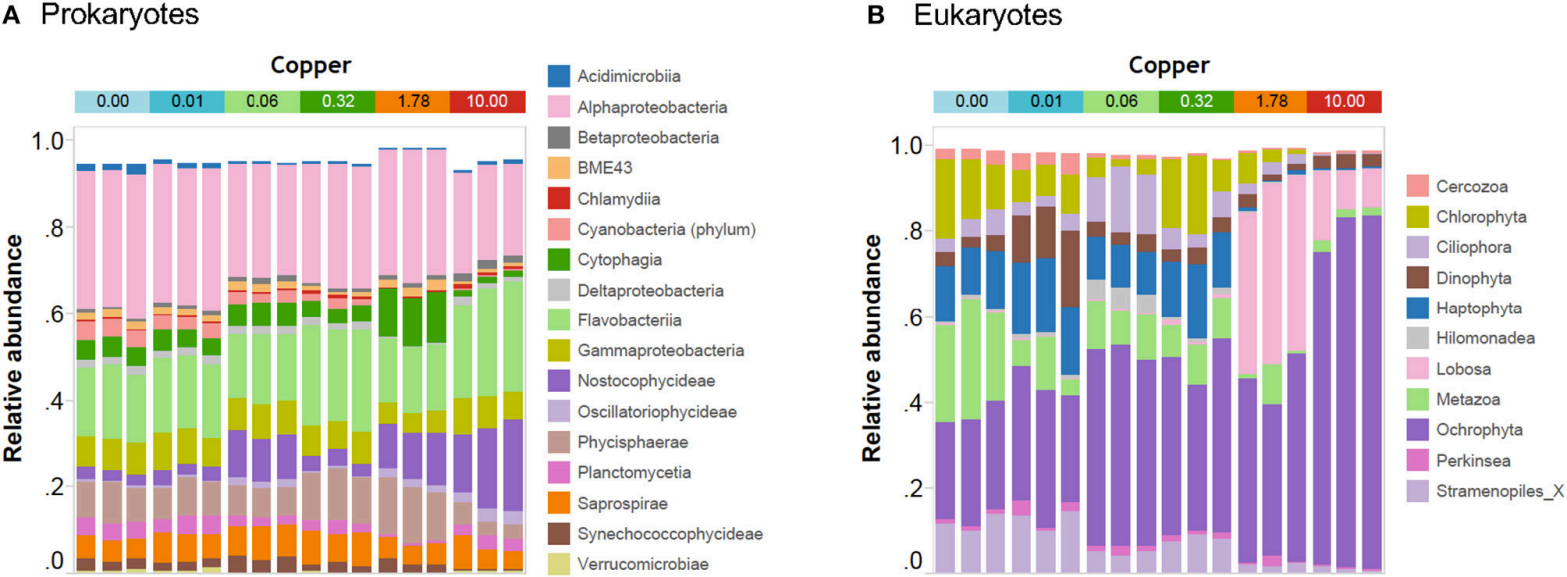
Bar-plot of the community composition of prokaryotic periphyton at class level **(A)** and of eukaryotic periphyton at phylum or higher taxonomic rank **(B)** recovered by amplicon-based high throughput sequencing. Note that Cu treatments: 0, 0.01, 0.06, 0.32, 1.78, and 10 are in μM Cu. Stramenopiles_X: refers to OTUs affiliated to other members of the Stramenopiles than Ochrophyta.

**Figure 3:**
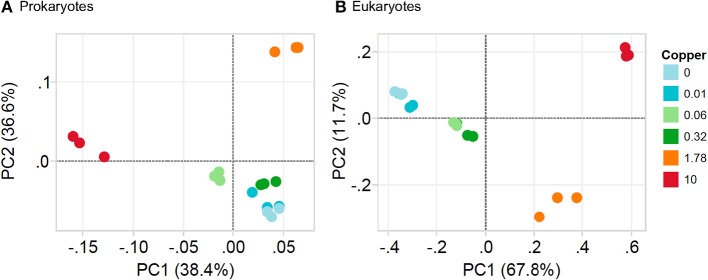
Principal component analysis (PCA) based on weighted UniFrac distances of OTUs composition of prokaryotic community **(A)** and eukaryotic community **(B)** in periphyton. Note that Cu treatments: 0, 0.01, 0.06, 0.32, 1.78, and 10 are in μM Cu.

**Figure 4:**
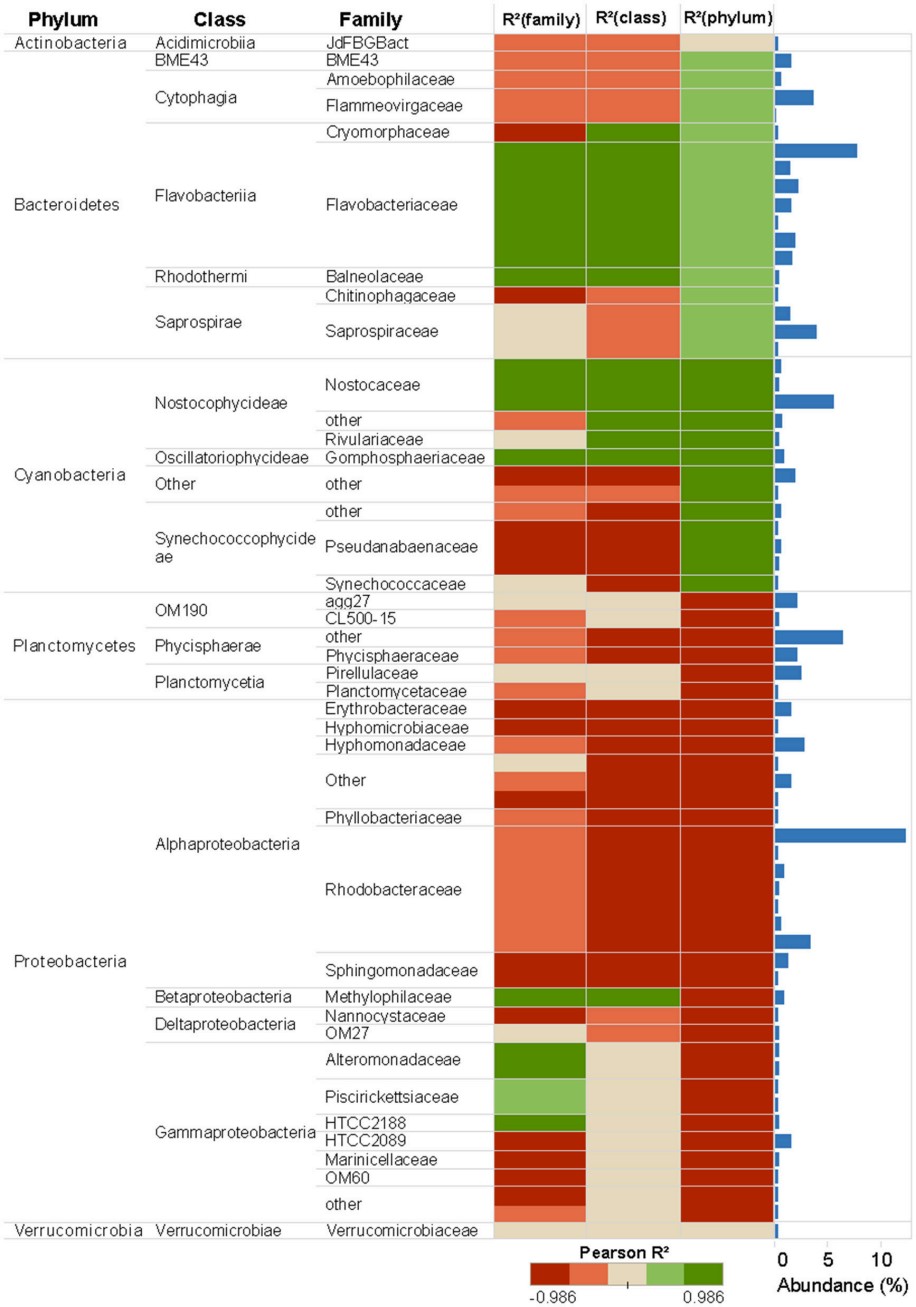
Prokaryotic abundance of taxa associated to 16S rRNA gene at different taxonomic levels (Phylum, Class, and Family) and Pearson correlations between Cu concentration and taxa abundance. Only the taxa whose reads number more than 2,000 were shown in the figure. Taxa abundance is presented in the bar plots to the right and include all the taxa detected in the sequence database.

In unexposed communities, the prokaryote community was dominated mainly by Alphaproteobacteria and Flavobacteria classes, and by taxa affiliated to Phycisphaerae and Saprosirae (Figure 2). The eukaryotic community was predominantly dominated by Ochrophyta and Metazoa, and by taxa affiliated with Chlorophyta, Haptophyta, and other members of Stramenopiles than Ochrophyta (Figure 2). Cu exposure decreased the total number of OTUs (Table 1) and also significantly reduced the chao1 diversity of both, the prokaryotic and the eukaryotic part of the periphyton (Supplementary Figure 1). The impacts of Cu on prokaryotic and eukaryotic community composition were confirmed by a MANOVA on Unifrac distances (Table 2). Significant changes of bacterial community composition were first observed after exposure to 0.06 μM Cu while the eukaryotic part of the periphyton was already significantly impacted after exposure to 0.01 μM Cu (Table 2, Figure 2). These findings are also reflected in the PCA plots that visualize the effects of Cu exposure on OTU abundance (Figure 3). For prokaryotes (Figure 3A), the first axis of the PCA explains 38% of the variance and grouped the samples from the control and 0.01, 0.06, 0.32, and 1.78 μM Cu on the right side of the axis, while samples from 10 μM Cu were grouped on the left side of the axis. The second axis of the PCA explained 37% of the variance and primarily separated the samples in three groups: (i) control and low levels of Cu exposure, from (ii) 1.78 μM Cu, and from (iii) 10 μM Cu (Figure 3A). The third axis was also significant and accounted for 12% with no clear separation of the samples. Remarkably, in treatments exposed to high levels of Cu (1.78 and 10 μM Cu), the relative abundance of prokaryotic sequences affiliated to Nostocophycideae and Oscillatoriophycideae classes were especially high and the relative abundance of sequences affiliated with Synechococcophycideae were especially low (Figures 2A, 4).

**Table 2:** Mean unifrac distance between Cu treatments.

	**Cu (μM)**	**0**	**0.01**	**0.06**	**0.32**	**1.78**	**10**
Prokaryote communities	0	0.0409					
	0.01	0.0637	0.0518				
	0.06	0.1193^*^	0.1034^*^	0.0330			
	0.32	0.1281^*^	0.1217^*^	0.1347^*^	0.0525		
	1.78	0.2107^*^	0.1983^*^	0.1895^*^	0.196^*^	0.0378	
	10	0.2127^*^	0.2028^*^	0.1663^*^	0.2092^*^	0.2413^*^	0.0574
Eukaryote communities	0	0.1296					
	0.01	0.3900^*^	0.1758				
	0.06	0.4496^*^	0.3794^*^	0.1249			
	0.32	0.4318^*^	0.4134^*^	0.2933^*^	0.1472		
	1.78	0.7605^*^	0.7346^*^	0.5675^*^	0.5112^*^	0.1637	
	10	0.9693^*^	0.9405^*^	0.7708^*^	0.7324^*^	0.5508^*^	0.0968

* *p < 0.001 by MANOVA test. Bold values stands for significant unifrac distance between Cu treatments*.

**Figure 5:**
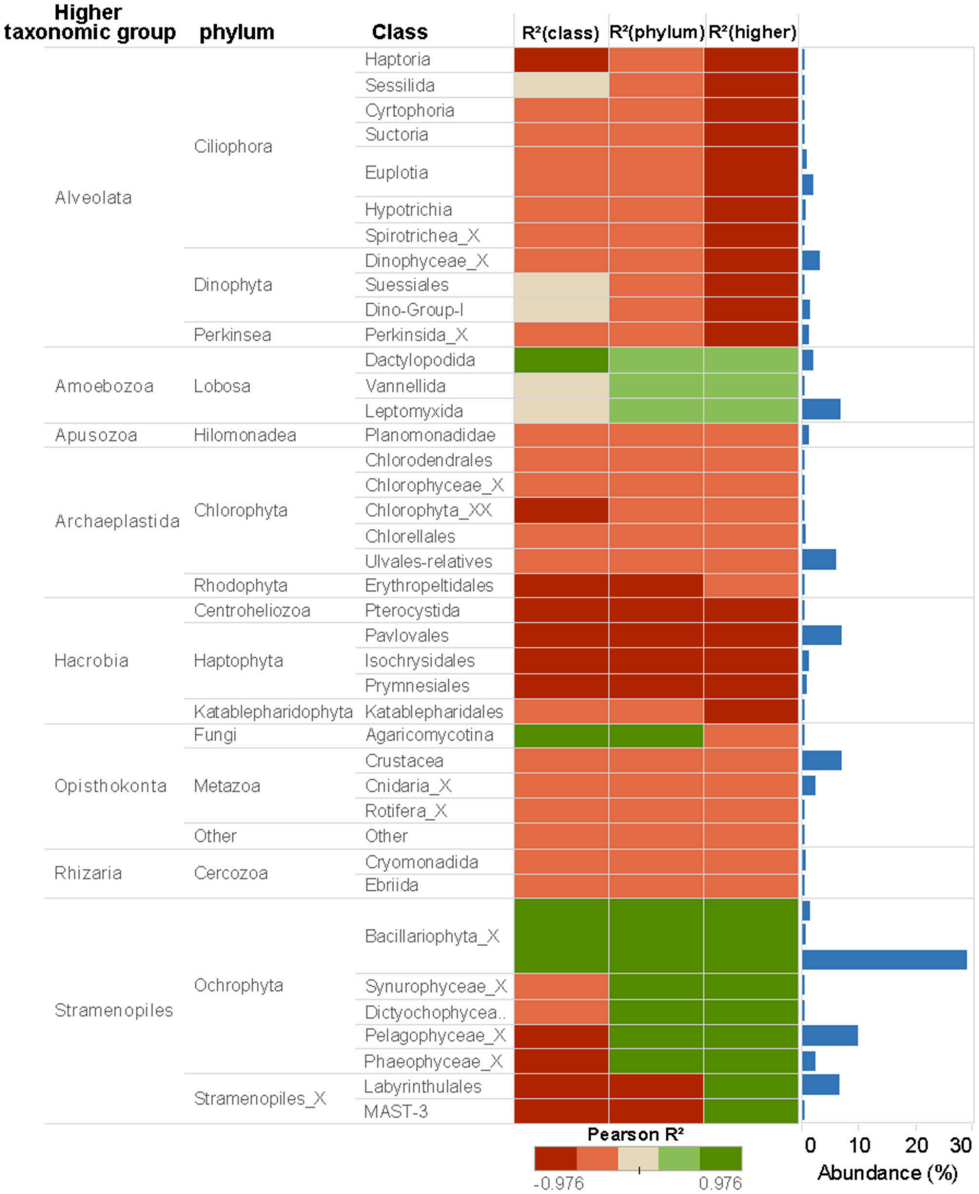
Eukaryotic abundance of taxa associated to 18S rRNA gene at different taxonomic levels (Class, Phylum or Higher Taxonomic Group) and Pearson correlations between Cu concentration and taxa abundance. Only the taxa whose reads number more than 2,000 were shown in the figure.

For Eukarytes, the first axis of the PCA explained 68% of the variance and separated the samples based on an increasing gradient of Cu exposure from the left side of the axis (control treatment) to the right site of the axis (highest Cu treatment, 10 μM Cu) (Figure 3B). The second axis explained circa 12% of the variance and it also separated the samples based on an increasing gradient of Cu exposure (Figure 3B). It should be pointed out that the relative abundance of sequences associated to Ochrophyta and Lobosa increased in treatments exposed to high levels of Cu (1.78 and 10 μM Cu; Figures 2A, 5). In contrast, the relative abundance of sequences associated to Haptophyta, Metazoa, Chlorophyt, Cliophora, Dinophyta, and Stramenoplies was markedly reduced when comparing to control treatment (Figures 2A, 5).

### Cu Sensitive and Tolerant Taxa and Changes in Community Network

Pearson correlation analyses showed different Cu sensitives among taxa (Figures 4, 5). As a general trend within prokaryotic taxa (i.e., at phylum level), the Cyanobacteria had a strong positive correlation with Cu exposure but, in contrast, abundances of Planctomycetes and Proteobacteria phyla had a strong negative correlation with Cu exposure (Figure 4). For eukaryotes, the Stramenopiles_X phylum was strongly positively correlated with Cu exposure and Amoebozoa was weakly positively correlated with Cu exposure. All other phyla within eukaryotes were negatively correlated to Cu exposure. In particular, Hacrobia and Alveolata showed a strong negative correlation to Cu exposure (Figure 5).

A total of 23 taxa can be classified as “sensitive,” showing a clear concentration-dependent decrease (Supplementary Figure 2 and Supplementary Table 2). For prokaryotes, the most sensitive taxa were from the phyla Proteobacteria and Bacteriodetes. Most of the sensitive taxa have a relatively high EC50 above 1 μM Cu, except for four taxa from the Cytophagales, Rickettsiales, Myxococcales and Oceanospirillales which had EC50 values ≤ 1 μM Cu (Supplementary Table 2). Five eukaryotic taxa from the Stramenopiles and Hacrobia taxonomic groups were sensitive to Cu, with EC50 values below 2 μM Cu (Supplementary Table 2).

**Figure 6:**
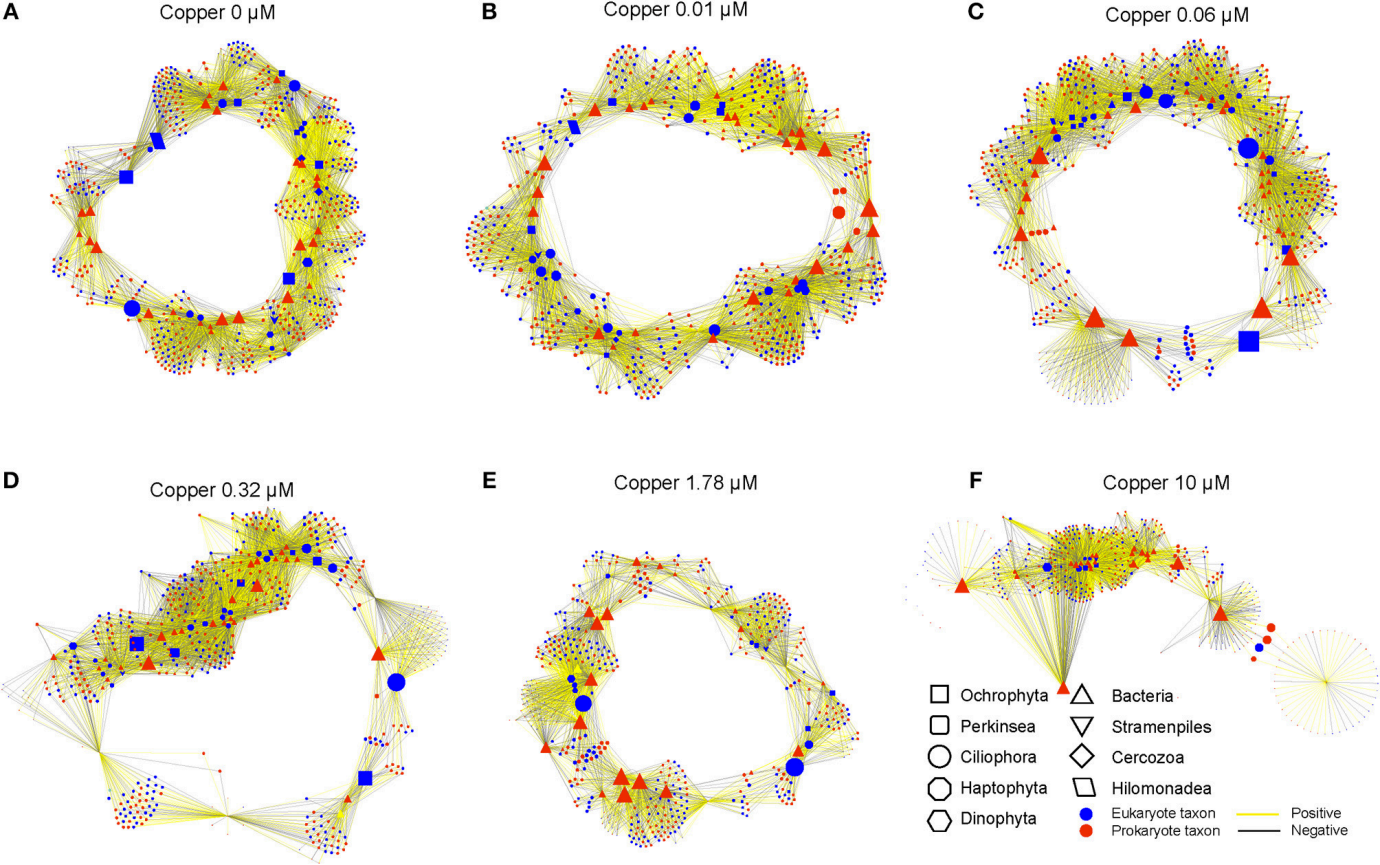
Network between prokaryotic and eukaryotic communities in different Cu exposure treatments: **(A)** (control), **(B)** (0.01 μM Cu), **(C)** (0.06 μM Cu), **(D)** (0.32 μM Cu), **(E)** (1.78 μM Cu), and **(F)** (10 μM Cu). Associations between taxa were generated by “Pearson” correlation analysis. Only correlations with a correlation >0.9 and a “two-tailed” *P* < 0.01 were reserved. Correlation coefficients between two nodes were labeled, the positive coefficient in yellow, while negative coefficient in black. The size of node indicates the “betweenness centrality”.

Cu changed the network of associations between taxa in the communities (Figure 6). In control and 0.01 μM Cu treatment, bio-interactions were domained by the Ciliophora, Dinophyta and Hilomonadea. However, bio-interactions were mainly domained by the Ciliophora in 1.78 and 10 μM of Cu. It's very interesting that in control and low Cu treatments, species are interrelated to form a closed circuit. When Cu concentration reaches 10 μM, the relationship between species becomes open linear. Low copper exposure increased the network associations, but at Cu concentrations of 0.32 μM and higher the number of nodes decreased and was lower than in the controls at 1.78 and 10 μM of Cu (Table 3).

**Table 3:** The topological parameters of network analysis.

	**0**	**0.01**	**0.06**	**0.32**	**1.78**	**10**
Number of nodes	517	529	525	506	453	402
Network heterogeneity	1.505	1.497	1.511	1.652	1.804	1.782
Network density	0.037	0.038	0.038	0.042	0.029	0.048
Muti-edge node pairs	234	252	282	225	104	232
Number of neighbors	19.277	20.276	19.73	21.083	13.223	19.139
Characteristic path length	3.454	3.403	3.488	3.43	3.507	3.728
Network centralization	0.231	0.26	0.24	0.302	0.277	0.368

## Discussion

Our microcosm study provides new insights into the ecological effects of long-term Cu exposure on marine prokaryotic and microeukaryotic organisms within periphyton biofilms. Cu exposure lasted 18 days, which is much longer than the generation time of studied microorganisms. Microbial prokaryotes and eukaryotes divide in a time range from hours to a few days. Hence, the applied exposure time is considered as long-term or chronic for our test system. Cu decreased prokaryotic and eukaryotic richness (number of OTUs) and the number of their interactions (number of nodes). Eukaryotes were more sensitive than prokaryotic taxa. Despite clear changes in the community structure, which rendered the exposed periphyton communities more Cu-tolerant, Cu exposure decreased the algal biomass and photosynthetic activity of exposed biofilms. Effects on community composition and function were observed at a Cu concentration of 0.06 μM, which is known to occur in the Swedish coastal environment (Egardt et al., 2018). The current environmental quality standard for Cu of 0.07 μM (HVMFS 2015:4) therefore does not seem to be sufficiently protective for periphyton, which contain key primary producers especially in coastal areas where the euphotic zone extends to the sediment (Wasmund, 1993; Sundbäck et al., 2004). Furthermore, it should be emphasized that the results were recorded in an ecologically realistic setting that allowed an ecological succession and competition to shape the communities under long-term chronic Cu exposure. Even though our study was performed in microcosms, prokaryotic and eukaryotic microbial composition established in our periphytic biofilms was comparable to that of *in situ* marine periphyton on artificial substrata from the same region (i.e., Gullmar fjord; Sanli et al., 2015; Corcoll et al., 2017).

The increase in pollution-induced community tolerance (PICT) coincided with changes in the structure and composition of the community (Figures 1–3), and it also coincided with a decrease in algal biomass and photosynthetic activity (Figure 1). This supports the view put forward first by Blanck et al. (1988), that community tolerance will increase as soon as sensitive species and genotypes are lost from the community. These observations are, however, in contrast to the functional redundancy hypothesis, which assumes that species loss has little impact on ecological functions (Oliver et al., 2015). Instead, they support the notion that biodiversity must be conserved fully, in order to ensure that an exposed community can continue to fulfill its ecological functions in a given ecosystem (Tilman and Downing, 1994). Although previous studies in biofilms have established a link between PICT and the structure of microbial organisms (Dorigo et al., 2010) or the genetic composition of photosynthetic micro-organisms (Eriksson et al., 2009a,b), this study is, to our best knowledge, the first paper that links PICT in biofilms with high-throughput DNA sequencing techniques (metabarcoding) targeting the whole prokaryotic and microeukaryotic microorganisms.

A wide range of sensitive prokaryotic and eukaryotic taxa was observed (Figures 4, 5). The highest Cu-tolerance amongst the prokaryotes was found in the Cyanobacteria phylum, especially in the Nostocophycideae and Oscilaltoriphycideae classes (Figure 4). Cyanobacteria resistance to Cu exposure has been observed previously (Barranguet et al., 2000; Serra et al., 2009) and might be attributed to their capacity to synthetize external ligands (Giner-Lamia et al., 2016) so that Cu is accumulated extracellularly (Serra et al., 2009).

Proteobacteria and Bacteroidetes phyla dominated the unexposed periphyton used in the present study, which was sampled from the Gullmar fjord on the Swedish west coast. This is in concordance with previous studies on marine bacterioplankton (Cottrell and Kirchman, 2000; Steven et al., 2012) and periphyton biofilms (Sanli et al., 2015; Corcoll et al., 2017). Cu tolerance of Bacteroidetes was observed in the present study with biofilms, but also similar paterns have been observed in Bacteroidetes from sediments exposed to Cu (Yang et al., 2018). In contrast, the Proteobacteria phylum was the most sensitive phylum to Cu. The abundance of 13 of its taxa, mainly from the dominant Alpha- and Gamma-proteobacteria classes, was reduced in a concentration-dependent manner, with EC50 values as low as 0.61 and 0.91 μM Cu, respectively (Supplementary Table 2). Many species of the phylum Proteobacteria are responsible for nitrification and denitrification processes, or linked with the assimilation of carbon (Ruiz-González et al., 2012; Sanli et al., 2015; Zhao et al., 2017). Given the high sensitivity of Proteobacteria to Cu, we therefore hypothesize that Cu pollution in marine areas could lead to impaired nitrogen cycles. Hence, in a further experiment it would be interesting to study impacts of Cu on microbial activities linked for example to N biogeochemical cycle (Larsson et al., 2007; Mußmann et al., 2013), and not only on photosynthesis.

Eight different eukaryotic higher taxonomic groups were detected (Alveolata, Amoebozoa, Apusozoa, Archaeplastida, Hacrobia, Opisthokonta, Rhizaria, and Stramenopiles; Figures 2, 5), demonstrating once again that benthic marine biofilms host a high biodiversity of prokaryote and microeukaryote organisms (Sanli et al., 2015; Corcoll et al., 2017). Stramenopiles and Amoebozoa were the most tolerant groups to Cu exposure, although the abundance of some fungal families within the Opisthokonta group (Figure 5). Bacillariophyta (diatoms), a family within the Stramenopiles group, was highly tolerant to Cu (Figure 5). Pigment analyses supported these results (Figure 1), since the relative abundance of fucoxanthin, a common marker for diatoms (Roy et al., 2011) also increased with increasing Cu concentrations. These findings agree with previous results of (Gustavson et al., 1999), who also reported an increase of centric Bacillariophyta in marine phytoplankton as a consequence of Cu exposure. The Cu tolerance in diatoms has been linked with their capacity to synthetize extracellular polysaccharides and frustuline (Gonçalves et al., 2018). Nevertheless, previously published mesocsom studies also provide a partly conflicting picture of diatom tolerance to Cu. For instance, in the studies by Barranguet et al. (2000) and Soldo and Behra (2000), diatoms from stream periphyton were less tolerant than green algae or cyanobacteria to long-term Cu exposure. Differences observed between these studies likely are explained because in each microcosm study a different starting algal community was used. Initial algal community composition has been described as a key factor for metal tolerance development in algal communities (Pérez et al., 2010).

In Fungi, the relative abundance of most classes and families was not affected by Cu exposure, which agrees with previous studies in sediment mesocosms (Gardham et al., 2014; Yang et al., 2018). Several resistance mechanisms in fungi to cope with Cu toxicity have been described, such as copper complexing by cell wall components, changes in membrane copper transport, synthesis of intra-cellular copper-binding metallothioneins and phytochelatins, and production of extracellular copper-complexing or -precipitating metabolites (Cervantes and Gutierrezcorona, 1994).

Five algal taxa where inhibited in a concentration-dependent manner: a member of the Pavlovaceae family (Haptophyta), a member of Erythropeltidales order (Rhodophyta) and three taxa within Stramenopiles, with EC50 values ranging from 1.2 to 2 μM Cu (Supplementary Table 2). The abundance of members of Hacrobia was strongly negatively correlated with Cu exposure (Figures 3, 5). Within Hacrobia, the relative abundance of its Haptophyta group declined with Cu exposure. Haptophyta is an important group in the oceans, especially calcifying Haptophyta (coccolithophores) which have a strong effect on the global carbon cycles (Tsuji and Yoshida, 2017). The abundance of other algal groups (Chlorophyta, Rhodophyta, and Dinophyta) was also reduced by Cu exposure, but only to a lower extent. We conclude that Cu effects on the aforementioned algal classes caused the observed decrease of total algal biomass and photosynthetic activity (Figure 1), which goes together well with previous studies that have demonstrated Cu toxicity to photosynthesis and algal growth at low concentrations (e.g., Pérez et al., 2010).

Ciliphora, a group of protozoa in the Alveolata superphylum, decreased in abundance under Cu exposure (Figure 5), which confirms previous results on the sensitivity of protozoa to Cu (Madoni et al., 1996; Yang et al., 2018). However, little is known about benthic protozoa in general. The use of DNA metabarcoding appears to be able to overcome these limitations and provide a new tool to investigate protozoa in ecotoxicological studies.

The abundances of most taxa from the Metazoa taxonomic group (Crustacea, Cnidaria, or Rotifera) were negatively correlated to Cu exposure (Figure 5). The sensitivity of Metazoa (i.e., Nematodes) to Cu and other environmental factors is well established (Bongers and Ferris, 1999; Boyd and Williams, 2003). This group of micro-eukaryotes eats particulate organic detritus, bacteria, algae, fungi, and protozoans. Hence, they act as regulators of decomposition and therefore play a key role in nutrient cycling and dynamics (Boyd and Williams, 2003; Stelzer, 2011). Hence, direct Cu effects on Metazoa may impact trophic interactions in periphyton biofilms, as network analysis indicate (Figure 6). The relative abundance of some diatoms (Bacillarophyta), within Ochrophyta phyla, increased with Cu exposure as shown by pigments and metabarcoding data (Figures 2B, 3, 5). However, Ochrophyta tend to disappear from the network analyses -with not anymore interactive nodes- when Cu exposure is increasing (Figure 6, Table 3). These results suggest that bacterial-algal interactions which are known to be important, especially in biofilms and microphytobenthos (Decleyre et al., 2015; Krohn-Molt et al., 2017), are negatively affected under Cu exposure. Overall, our network analyses demonstrate that Cu changed the associations between various taxa in the prokaryotic and eukaryotic communities (Figure 6, Table 3) suggesting that the trophic chain interactions and the microbial loop in periphyton biofilms will be altered under certain levels of Cu exposure.

Even though DNA metabarcoding has emerged as a prominent technique to detect a large number of taxa in an environmental sample (Hebert et al., 2003), the technique also has its limitations. The choice of primers affects the biodiversity assessment, and a perfectly universal primer is difficult or even impossible to design (Klindworth et al., 2013; Hugerth et al., 2014; Zhang et al., 2018). To overcome these limitations, a combination of many specific primers to target each of the eukaryotic kingdoms (e.g., ITS gene to target fungi; Nilsson et al., 2009, 23S gene to target algae; Sherwood et al., 2008, or COI to target invertebrates; Leray and Knowlton, 2015) might provide an improved resolution and less bias. In this study, we chose the V3 region of the 16S rRNA gene to target bacteria and the V9 region of the 18S rRNA to target eukaryotes. Both regions are widely used in DNA metabarcoding of microbial communities in various ecosystems (Amaral-Zettler et al., 2009; Klindworth et al., 2013; Corcoll et al., 2017; Yang et al., 2018). However, the region V4 of the 18S rRNA gene has been suggested as an alternative to the V9 region, in order to capture more diversity (Pernice et al., 2013; Hugerth et al., 2014). Another limitation of current metabarcoding approaches, specifically with respect to marine microbial communities is the low coverage in public sequence repositories for many natural microorganisms, and especially microeukaryotes (Bik et al., 2012; Sanli et al., 2015). In spite of the incompleteness of DNA reference libraries, the suitability of metabarcoding for ecological assessment in freshwater ecosystems, using benthic diatoms, has been recently demonstrated (Rivera et al., 2018).

To conclude, this study allowed us to detected changes in the community composition of benthic pro- and eu-karyotes already at 0.06 and 0.01 μM Cu, respectively. These effect concentrations are environmentally realistic (Egardt et al., 2018) and are below the current environmental quality standards (EQS) for copper on the Swedish west coast (HVMFS, 2015). Hence, observed mesocosm results suggest that the current Cu EQS for the marine environment are not protective for prokaryotic and eukaryotic microbial organisms in marine biofilms. Our results provide new information of how Cu pollution affects microbial biodiversity and community composition in the marine environment, data that will aid the setting of appropriate environmental quality standards. Furthermore, this work shows the robustness and the promising potential of DNA metabarcoding as a sensitive tool for community-level ecotoxicological studies that allows to observe impacts simultaneously on a multitude of pro- and eukaryotic taxa, and therefore to identify particularly sensitive, non-cultivable taxa.

## Author Contributions

NC, KME, and TB designed the work. NC and KME performed the experiment, processed the samples and performed the analyses for non-sequencing data. NC extracted the DNA. Amplicon sequencing and bioinformatic analyses were performed by JY and XZ. NC wrote the first draft of the paper. All authors discussed, interpreted the results, and contributed to producing the paper.

### Conflict of Interest Statement

The authors declare that the research was conducted in the absence of any commercial or financial relationships that could be construed as a potential conflict of interest.

## Supplementary Material

Supplementary Figure S1 Average ± standard deviation (n = 3) of chao1 index in each copper treatment (0, 0.01, 0.06, 0.32, 1.78 and 10 μM Cu) in function of number of sequences associated to 16S rRNA gene **(A)** or to the 18S rRNA gene **(B)**.

Supplementary Figure S2 Concentration response curves for the 23 taxa identified as copper sensitive, their EC50s, standard deviation of EC50s. The taxonomy assignment of each taxon was showed in Supplementary Table 2.
