# Supplementary material for: Copper Affects Composition and Functioning of Microbial Communities in Marine Biofilms at Environmentally Relevant Concentrations
Source: Front Microbiol. 2019 Jan 8;9:3248. doi: 10.3389/fmicb.2018.03248 (PMC6331542; doi:10.3389/fmicb.2018.03248)
Supplement: Supplementary file 1 [file Table_1.docx]

Supplementary Table S1. Nominal and analysed copper concentrations (AVG ± SD; n=3) in the microcosms before and after the water renewals. The average copper concentration between before and after water renewals is also presented, as well as the percentage of analysed copper concentrations versus the nominal concentrations. Note: LoQ = 0.02 µM

|  |  |  |  |  |
| --- | --- | --- | --- | --- |
| Nominal conc. (µM Cu) | Before water renewal  (µM Cu) | After water renewal  (µM Cu) | Average conc. before and after water renewals | |
|  |  |  | µM Cu | Analyzed as % of nominal conc. |
| 0 | < LoQ | < LoQ | < LoQ | - |
| 0.01 | < LoQ | < LoQ | < LoQ | - |
| 0.06 | 0.02 ± 0.002 | 0.02 ± 0.001 | 0.02 | 33 |
| 0.32 | 0.19 ± 0.07 | 0.15 ± 0.01 | 0.17 | 53 |
| 1.78 | 1.43 ± 0.51 | 1.61 ± 0.20 | 1.5 | 84 |
| 10 | 7.88 ± 1.84 | 8.26 ± 0.88 | 8.1 | 81 |
