# Supplementary material for: Copper Affects Composition and Functioning of Microbial Communities in Marine Biofilms at Environmentally Relevant Concentrations
Source: Front Microbiol. 2019 Jan 8;9:3248. doi: 10.3389/fmicb.2018.03248 (PMC6331542; doi:10.3389/fmicb.2018.03248)
Supplement: Supplementary file 2 [file Table_2.docx]

Supplementary Table S2. The taxonomic affiliation, EC50 values (AVG) and their standard deviation (SD) in µM Cu, for the taxa classified as sensitive to Cu.

| Taxon | Taxa | EC50 (AVG) | (SD) |
| --- | --- | --- | --- |
| 1 | k__Bacteria;p__BRC1;c__PRR-11;o__;f__ | 1.5 | 0.81 |
| 2 | k__Bacteria;p__Bacteroidetes;c__Cytophagia;o__Cytophagales;f__Cytophagaceae | 1.0 | 0.34 |
| 3 | k__Bacteria;p__Bacteroidetes;c__Flavobacteriia;o__Flavobacteriales;Other | 1.9 | 2.5 |
| 4 | k__Bacteria;p__Bacteroidetes;c__Flavobacteriia;o__Flavobacteriales;f__Cryomorphaceae | 9.8 | 4.5 |
| 5 | k__Bacteria;p__Chlamydiae;c__Chlamydiia;o__Chlamydiales;Other | 1.8 | 0.74 |
| 6 | k__Bacteria;p__Proteobacteria;c__Alphaproteobacteria;Other;Other | 1.9 | 0.78 |
| 7 | k__Bacteria;p__Proteobacteria;c__Alphaproteobacteria;o__Rhodobacterales;Other | 5.4 | 2.5 |
| 8 | k__Bacteria;p__Proteobacteria;c__Alphaproteobacteria;o__Rickettsiales;f__ | 0.94 | 0.47 |
| 9 | k__Bacteria;p__Proteobacteria;c__Alphaproteobacteria;o__Sphingomonadales;Other | 8.9 | 1.6 |
| 10 | k__Bacteria;p__Proteobacteria;c__Alphaproteobacteria;o__Sphingomonadales;f__ | 8.5 | 3.7 |
| 11 | k__Bacteria;p__Proteobacteria;c__Alphaproteobacteria;o__Sphingomonadales;f__Erythrobacteraceae | 7.1 | 2.1 |
| 12 | k__Bacteria;p__Proteobacteria;c__Deltaproteobacteria;o__Myxococcales;f__Nannocystaceae | 0.91 | 0.37 |
| 13 | k__Bacteria;p__Proteobacteria;c__Gammaproteobacteria;o__Alteromonadales;f__OM60 | 6.0 | 1.1 |
| 14 | k__Bacteria;p__Proteobacteria;c__Gammaproteobacteria;o__HTCC2188;f__HTCC2089 | 2.0 | 0.39 |
| 15 | k__Bacteria;p__Proteobacteria;c__Gammaproteobacteria;o__Legionellales;f__ | 1.6 | 0.63 |
| 16 | k__Bacteria;p__Proteobacteria;c__Gammaproteobacteria;o__Oceanospirillales;Other | 9.5 | 1.1 |
| 17 | k__Bacteria;p__Proteobacteria;c__Gammaproteobacteria;o__Oceanospirillales;f__ | 0.61 | 0.11 |
| 18 | k__Bacteria;p__Proteobacteria;c__Gammaproteobacteria;o__Marinicellales;f__Marinicellaceae | 6.2 | 2.0 |
| 19 | k_Eukarya; Archaeplastida(no rank);Rhodophyta(no rank);c__Compsopogonophyceae;o__Erythropeltidales;f__Erythropeltidales_X | 2.0 | 1.5 |
| 20 | k_Eukarya; Hacrobia(no rank);Haptophyta(no rank);Pavlovophyceae(no rank);o__Pavlovales;f__Pavlovaceae | 1.2 | 3.7 |
| 21 | K_Eukarya; p_Stramenopiles; Stramenopiles_X;_c_Labyrinthulea;f__Thraustochytriales;g__Thraustochytriaceae | 2.0 | 4.1 |
| 22 | k_Eukarya; p_Stramenopiles; c_Stramenopiles_X;MAST;MAST-3;MAST-3J | 1.8 | 0.95 |
| 23 | p__Stramenopiles;Stramenopiles_X;Other;Other;Other | 1.8 | 0.29 |
