# Supplementary figures and images for: Copper Affects Composition and Functioning of Microbial Communities in Marine Biofilms at Environmentally Relevant Concentrations
Source: Front Microbiol. 2019 Jan 8;9:3248. doi: 10.3389/fmicb.2018.03248 (PMC6331542; doi:10.3389/fmicb.2018.03248)

**A**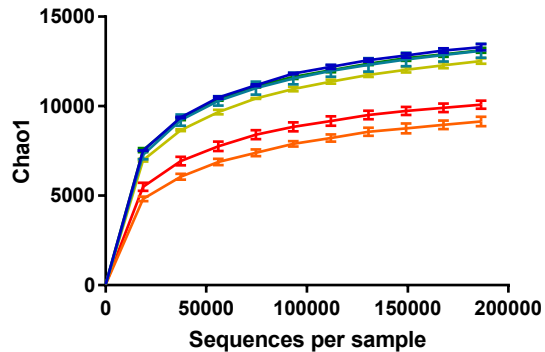**B**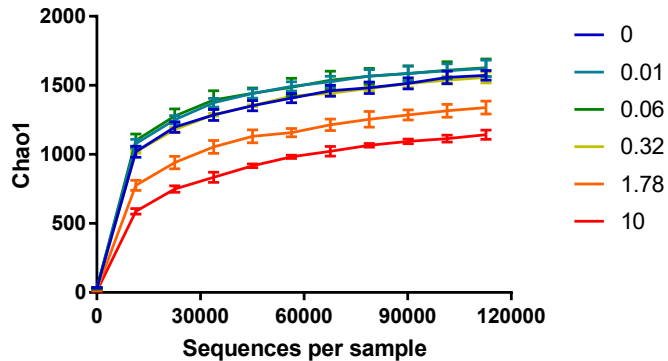

Supplement: Supplementary Figure S1 — Average ± standard deviation (n = 3) of chao1 index in each copper treatment (0, 0.01, 0.06, 0.32, 1.78 and 10 μM Cu) in function of number of sequences associated to 16S rRNA gene (A) or to the 18S rRNA gene (B). [file Image_1.PDF]

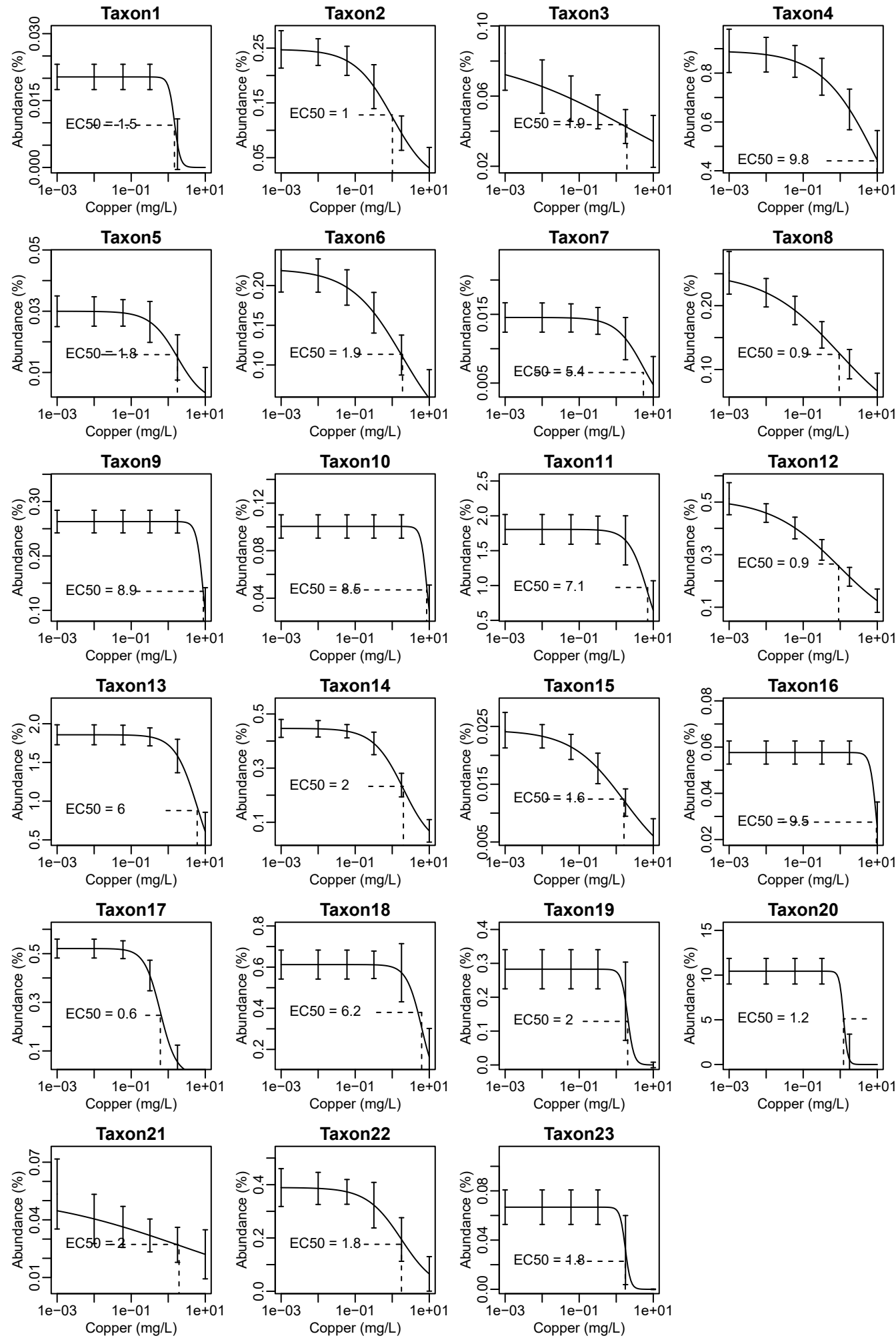

Supplement: Supplementary Figure S2 — Concentration response curves for the 23 taxa identified as copper sensitive, their EC50s, standard deviation of EC50s. The taxonomy assignment of each taxon was showed in Supplementary Table 2. [file Image_2.PDF]
